# An Unusual Cause of Cast Nephropathy in a Patient Presenting With Hypercalcemia and Renal Failure: A Case Report

**DOI:** 10.7759/cureus.101350

**Published:** 2026-01-12

**Authors:** Taif Najeebi, Shruti Prem Sudha, Fatima Al-Hashimi, Mahera Roohi, Salwa Ali

**Affiliations:** 1 Department of Hematology, Bahrain Oncology Center, Muharraq, BHR; 2 Department of Pathology, Blood Bank and Laboratory Medicine, King Hamad University Hospital, Muharraq, BHR; 3 Department of Radiology, King Hamad University Hospital, Muharraq, BHR

**Keywords:** cast nephropathy, hypercalcemia, non-oliguric renal failure, petct, sarcoidosis

## Abstract

Sarcoidosis is a multisystem disease that is typically characterized by end-organ damage due to the formation of non-necrotizing granulomas in affected tissues. The lungs and lymph nodes are the most commonly affected organs; however, almost any organ can be involved. The diagnosis of sarcoidosis is based on a compatible clinical and radiological presentation, as well as histopathological evidence of non-necrotizing granulomas in affected organs. The diagnosis can be challenging, given the variability in clinical presentation and organ involvement, especially in cases without pulmonary symptoms.

Renal involvement in sarcoidosis is complex and is usually attributed to nephrocalcinosis or damage to the tubulointerstitium of the kidney caused by granulomas. Cast nephropathy, which results from injury to the renal tubules due to the toxic effects of immunoglobulin light chains excreted in the urine, has previously been described in renal sarcoidosis and is most often reported in the context of hematological malignancies such as myeloma or lymphoma. We report the case of a patient who presented with renal dysfunction, hypercalcemia, and albumin-globulin (A/G) reversal mimicking multiple myeloma, but further investigations, including imaging and tissue biopsy, ultimately established a diagnosis of sarcoidosis. This report highlights the importance of a comprehensive diagnostic approach in such multisystem disorders.

## Introduction

Cast nephropathy is a severe condition in which monoclonal light chains (abnormal proteins secreted by plasma cells) precipitate in the renal tubules and interact with Tamm-Horsfall protein to form obstructive plugs (casts). The obstruction of distal and collecting tubules causes tubular injury and atrophy and triggers an inflammatory response, which can lead to interstitial nephritis and fibrosis. It is a defining feature of multiple myeloma but can occasionally be seen in other conditions such as B-cell lymphoproliferative disorders [1,2].

We present a case involving a rare cause of cast nephropathy in a patient who presented with renal dysfunction due to light chain cast nephropathy, hypercalcemia, and albumin-globulin (A/G) reversal that mimicked multiple myeloma. Further investigations, including imaging and tissue biopsy, ultimately established the diagnosis of sarcoidosis. Sarcoidosis is a multisystem disease of unknown origin characterized by the formation of granulomas in various organs [3]. The diagnosis of sarcoidosis is based on clinical and radiological criteria, as well as histopathological evidence of non-necrotizing granulomatous inflammation in affected organs. Renal involvement in sarcoidosis is multifactorial and is usually due to nephrocalcinosis, tubulointerstitial nephritis from granulomas, or, less commonly, glomerulonephritis [4]. Cast nephropathy, as observed in our patient, has not previously been reported in renal sarcoidosis.

As seen in our patient, the diagnosis of sarcoidosis can sometimes be challenging, especially in atypical presentations, due to variability in the organs involved. Hypercalcemia may provide a valuable clue to the diagnosis, particularly in cases with no pulmonary symptoms or findings on chest X-ray. This report emphasizes the importance of a comprehensive diagnostic approach in such multisystem disorders, which often have overlapping clinical features.

## Case presentation

A 59-year-old male presented to the emergency department with generalized fatigue, weight loss of 5 kg over the last four months, and lower back pain, suggestive of bone pain for the last six months. He gave a history of discoloration and ulceration of finger tips, and hyperkeratosis of skin over arms and legs. He had recently been found to have worsening renal function over the past month. Clinical examination revealed a cachectic patient with pallor, digital ulcers, and blackish discoloration of finger tips resembling cutaneous necrosis, hyperkeratotic, dry skin over the forearms and legs, and palpable splenomegaly.

Preliminary blood workup (Table 1) revealed normocytic anemia, high calcium levels of 2.81 mmol/L (11.3 mg/dL), lactate dehydrogenase (LDH) of 107 U/L, and creatinine of 472.1 μmol/L (5.3 mg/dL). Urine analysis showed protein of 100 mg/dL, and 24-hour urine protein was 1.24 g/L. Antinuclear antibody (ANA) profile was negative, and the angiotensin-converting enzyme level was normal. The patient's total serum protein level was 82.9 g/L, with high globulin of 43 g/L, and A/G reversal was noted. Quantification of serum immunoglobulin levels showed a high IgG level of 3185.5 mg/dL (normal range: 681-1648 mg/dL), with normal levels of IgA of 407 mg/dL(normal range: 87-474 mg/dL) and low IgM of 38.6 mg/dL(normal range: 48-312 mg/dL). Serum protein electrophoresis was indicative of a polyclonal increase in gamma globulin, immune-fixation was negative for a monoclonal band, and the free light chains showed a polyclonal increase with a normal kappa-lambda ratio (free kappa was 2.45 mg/dL, free lambda was 2.57mg/dL, and the kappa-lambda ratio was 0.96).

**Table 1:** Laboratory values at presentation

Parameter	Baseline values	Reference range
Hemoglobin	7.9 g/dL	13–16 g/dL
Mean corpuscular volume	78.8 fL	80–100 fL
Serum calcium	2.81 mmol/L	2.12–2.62 mmol/L
Lactate dehydrogenase	107 U/L	100–190 U/L
Serum creatinine	472.1 µmol/L	62–115 µmol/L
Urine protein	100 mg/dL	0
24-hour urine protein	1.24 g/L	<0.05–0.1 g/L
Antinuclear antibody	Negative	Negative
Angiotensin-converting enzyme	37 U/L	12–68 U/L
Total serum protein	82.9 g/L	60–83 g/L
Globulin	43 g/L	20–35 g/L
Albumin/globulin ratio	0.41	>1.0
Immunoglobulin G	3185.5 mg/dL	681–1648 mg/dL
Immunoglobulin A	407.3 mg/dL	87–474 mg/dL
Immunoglobulin M	38.6 mg/dL	48–312 mg/dL
Total protein	76.5 g/L	64–82 g/L
Albumin	33.2 g/L	40–47 g/L
Alpha-1 globulin	4.9 g/L	2.1–3.5 g/L
Alpha-2 globulin	6.8 g/L	5.1–8.5 g/L
Beta-1 globulin	5.3 g/L	3.1–6.5 g/L
Beta-2 globulin	5.1 g/L	2.3–4.7 g/L
Gamma globulin	23 g/L	8–13.5 g/L
Free kappa light chain	2.45 mg/dL	0.33–1.94 mg/dL
Free lambda light chain	2.57 mg/dL	0.57–2.60 mg/dL
Kappa-lambda ratio	0.96	0.26–1.65
Monoclonal band	Not detected	Not detected
Immunotyping	Normal	Normal
M-Spike	Not detected	Not detected

Based on the clinical picture of A/G reversal, renal dysfunction, proteinuria, and hypercalcemia, it was decided to proceed with further evaluation for multiple myeloma. Bone marrow aspiration and biopsy were performed, which showed reactive plasmacytosis of 10-15% with no evidence of clonality, as there was no light chain restriction on immunohistochemistry or flow cytometry.

**Figure 1:**
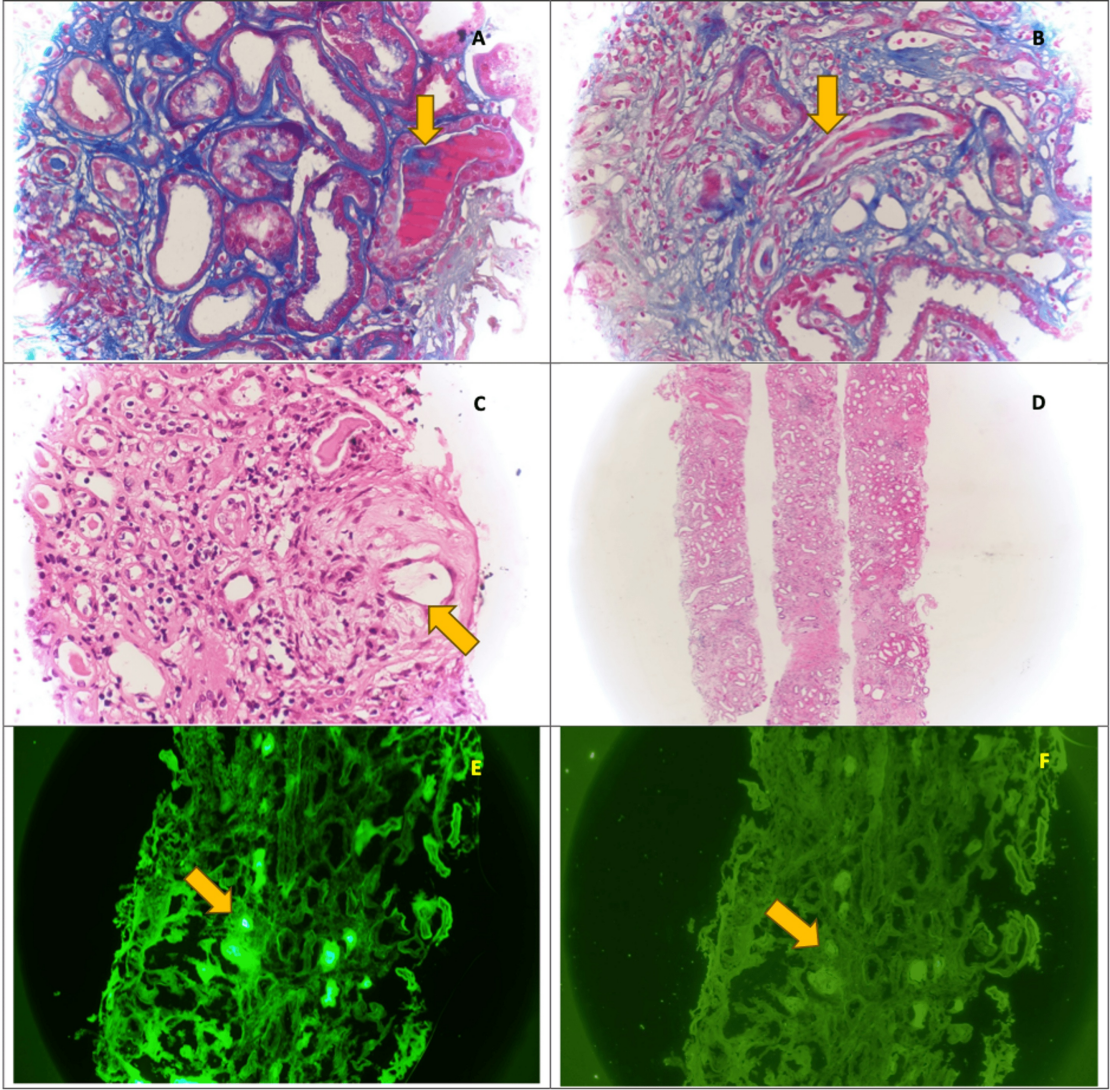
Renal biopsy findings (A) Polychromatic casts in the tubules, arrow points to the cast with variation in color between blue and red, indicating possible monoclonality, appearing fractured in (B), injuring and rupturing the lumina in (C); the arrow points to tubular injury with surrounding inflammatory response. Zonal interstitial fibrosis and tubular atrophy in the background (D), no granulomatous inflammation or amyloidosis. On immunofluorescence, the tubular casts are highlighted by lambda (E), while negative on kappa (F), confirming monoclonality; the arrow shows bright luminous staining of the casts in E, while they are dull in F

Given the clinical suspicion of amyloidosis due to renal dysfunction, proteinuria, and the presence of cutaneous manifestations, a renal biopsy was performed. This revealed light chain (lambda-restricted) cast nephropathy, mild-to-moderate diabetic glomerulopathy, and diabetic nephropathy (Figure 1). No evidence of amyloidosis was identified on light microscopy, immunofluorescence, or electron microscopy. There was also no evidence of nephrocalcinosis, granulomas, or immune complex deposition in the glomeruli.

**Figure 2:**
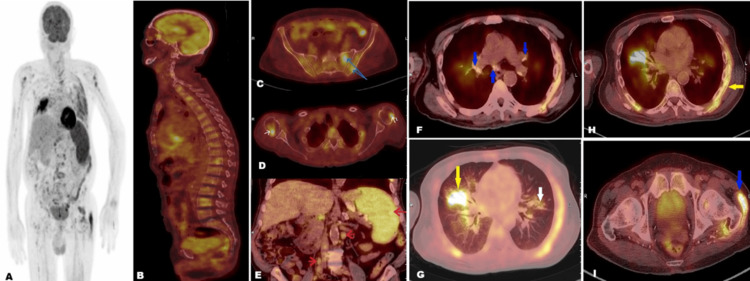
PET-CT findings Whole-body FDG PET images. Maximum intensity projection (A) and fused PET-CT images (B-I) showing patchy marrow activity in spines, pelvic bones (blue arrows in panel C) and humeri (white arrows in panel D), splenomegaly with enhanced FDG activity and active FDG-avid abdominal lymph nodes (red arrows in panel E), inactive calcified mediastinal nodes (blue arrows in panel F), right middle lung lobe consolidation (yellow arrow in panel G), peri-fissural nodular infiltrates (white arrow in panel G), myositis ossificans at left chest wall (yellow arrow in panel H) and at lateral aspect of left femoral trochanter (blue arrow in panel I) PET-CT: positron emission tomography/computed tomography; FDG: fluorodeoxyglucose

In light of the finding of cast nephropathy, a PET-CT was requested to rule out lymphoproliferative disorders (Figure 2). This revealed FDG-avid splenomegaly, patchy marrow-based skeletal FDG uptake, multiple FDG-avid extra-skeletal calcified sheets suggestive of metastatic calcification, and FDG-avid pulmonary interstitial and nodular infiltrates.

**Figure 3:**
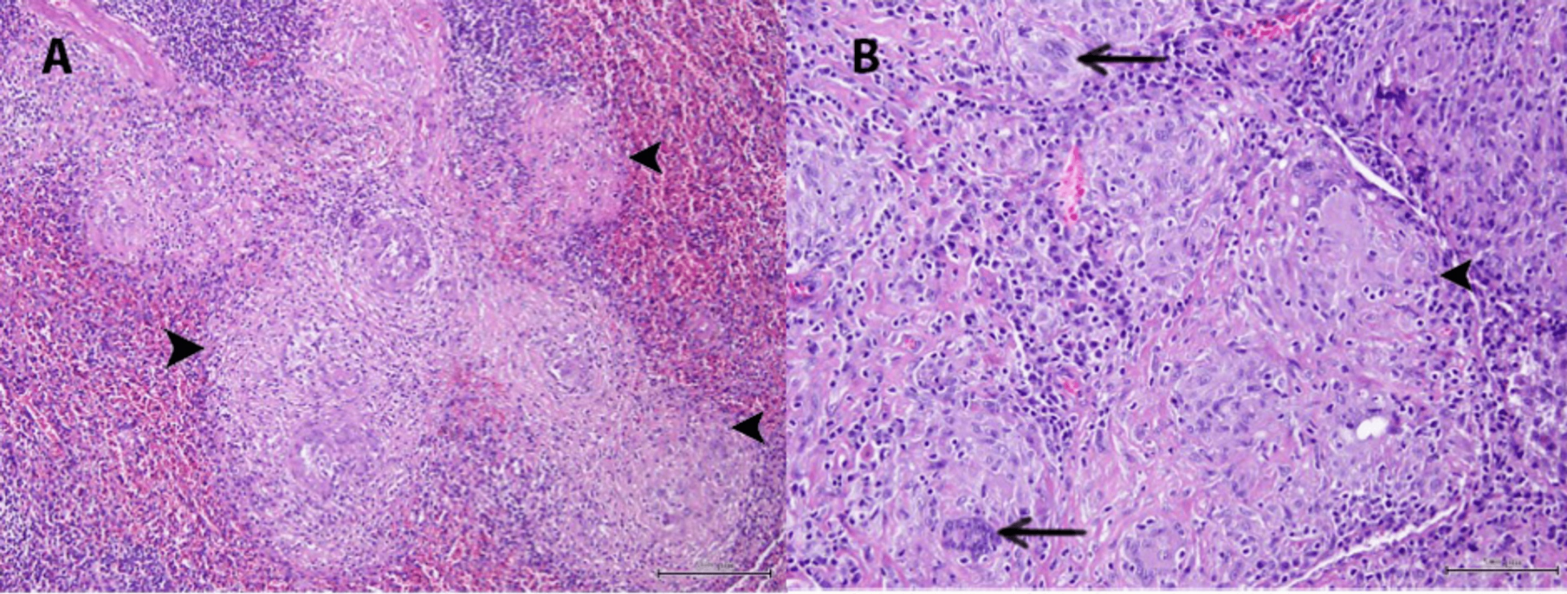
Histopathological examination of the spleen Low-power (A) and high-power (B) images of splenic tissue. The arrows in panel A point to the numerous non-caseating epithelioid cell granulomas, and the arrows in panel B point to the giant cells; the overall picture is consistent with sarcoidosis

Autoimmune workup was negative for rheumatological disorders. Doppler of the distal arteries and arterioles in hands and feet did not show any compromise of blood flow. Given that all workup to date had not yielded a definitive diagnosis, a decision was made to proceed with a splenectomy for tissue diagnosis based on the observed hypermetabolic enlarged spleen on PET-CT. Histopathological examination of the spleen (Figure 3) showed extensive involvement by numerous non-caseating epithelioid cell granulomas and giant cells consistent with sarcoidosis. The involved splenic tissue showed areas of eosinophilic material deposition, negative for Congo-red stain. The excised lymph nodes also showed similar granulomatous inflammation. In view of the clinical profile and histology consistent with sarcoidosis, treatment with intravenous corticosteroids was initiated, leading to improvement in renal function, and the patient was transferred to the rheumatology team for further treatment.

## Discussion

This report highlights a rare presentation of sarcoidosis mimicking multiple myeloma. Sarcoidosis is a multisystem disease characterized by non-caseating granulomas affecting a variety of organs, typically with pulmonary and lymphatic involvement, with renal manifestations being much rarer. Sarcoidosis can present with atypical features that mimic other diseases, leading to diagnostic challenges [5,6]. Our patient presented with stereotypical features of myeloma, including anemia, renal dysfunction, bone lesions, hypercalcemia, and A/G reversal. A similar presentation has been reported by El-Husseini et al. [7], who described a patient with sarcoidosis diagnosed during renal workup for possible myeloma; here, the sarcoidosis was diagnosed through the presence of non-caseating granulomas in the bone marrow biopsy, while renal biopsy revealed mesangial proliferative glomerulonephritis with significant mesangial IgG deposition.

Impaired renal function in sarcoidosis has been attributed to either hypercalcemia with nephrocalcinosis and renal calculi, or to granulomatous involvement of renal parenchyma and tubulointerstitial disease secondary to hypercalcemia and granulomatous disease [8,9]. Primary glomerular alterations are not generally associated with sarcoidosis; however, rare cases have reported glomerular pathologies such as immune complex deposition, IgM-associated glomerulonephritis, and mesangial IgG deposition resulting in mesangioproliferative glomerulonephritis [7,10,11]. In a study of 47 patients with renal sarcoidosis, the authors reported non-caseating granulomatous interstitial nephritis (GIN) in the majority (37 patients), while ten patients had interstitial nephritis without granuloma [12].

In our patient, no granulomas were observed, and the main finding was that of tubular inflammation secondary to cast nephropathy, and the casts were lambda light chain-restricted. Cast nephropathy has not been previously reported in the setting of renal sarcoidosis, though coincidentally occurring monoclonal gammopathy has been described [7]. The urine immune-fixation in our patient showed proteinuria without any monoclonal peaks in the IgG, IgM, and IgA regions or in the kappa and lambda regions. In our patient, the casts were composed of lambda light chain, but the blood test showed polyclonal rather than monoclonal hypergammaglobulinemia; hence, the precise causative factor in the development of the cast nephropathy cannot be determined.

Sarcoidosis may be complicated by systemic vasculitis involving small- to large-caliber vessels, and this was initially considered to explain the digital ulcerative lesions observed. In our patient, no vascular compromise was identified in the distal extremities on Doppler imaging; however, capillary or arteriolar disease may not be detectable by Doppler. It is possible that the digital lesions were a consequence of calcinosis cutis, which has been reported to cause ulceration and cutaneous gangrene in severe cases. This explanation is supported by PET-CT findings demonstrating extensive subcutaneous calcification; however, a skin biopsy would have provided more definitive confirmation, which was not performed in our patient. A wide range of cutaneous manifestations have been reported in sarcoidosis [5,6], including erythema nodosum, lupus pernio, plaques, patches, and nodules. Calcinosis cutis leading to ulceration has also been described, albeit less commonly [5,6].

## Conclusions

Clinicians and internists should consider sarcoidosis in the differential diagnosis of patients with multisystem involvement, particularly those presenting with renal dysfunction and cutaneous manifestations, even in the absence of pulmonary involvement. This case highlights the diverse and often atypical clinical presentations of sarcoidosis and emphasizes the need for a holistic diagnostic approach in such cases. To our knowledge, this is the only reported case of cast nephropathy in renal sarcoidosis. Long-term follow-up will help clarify the subsequent course of nephropathy and the response to sarcoidosis-directed therapy in our patient.
